# Chia Phenolic Extract Appear to Improve Small Intestinal Functionality, Morphology, Bacterial Populations, and Inflammation Biomarkers In Vivo (*Gallus gallus*)

**DOI:** 10.3390/nu15163643

**Published:** 2023-08-19

**Authors:** Marcella Duarte Villas Mishima, Hércia Stampini Duarte Martino, Nikolai Kolba, Nikita Agarwal, Cydney Jackson, Bárbara Pereira da Silva, Mariana Grancieri, Andressa de Assis, Vinícius Parzanini Brilhante de São José, Elad Tako

**Affiliations:** 1 Department of Food Science, Stocking Hall, Cornell University, Ithaca, NY 14853, USA; md983@cornell.edu (M.D.V.M.); nk598@cornell.edu (N.K.); na494@cornell.edu (N.A.); cdj53@cornell.edu (C.J.); 2 Department of Nutrition and Health, Federal University of Viçosa, Viçosa 36570-900, MG, Brazil; hercia@ufv.br (H.S.D.M.); barbara.p.silva@ufv.br (B.P.d.S.); marianagrancieri@gmail.com (M.G.); andressa.assis@ufv.br (A.d.A.); vinicius.sao@ufv.br (V.P.B.d.S.J.)

**Keywords:** *Salvia hispanica* L., phenolic compounds, intra-amniotic administration, intestinal health, BBM

## Abstract

Phenolic compounds can act as a substrate for colonic resident microbiota. Once the metabolites are absorbed and distributed throughout the body, they can have diverse effects on the gut. The objective of this study was to evaluate the effects of the intra-amniotic administration of a chia phenolic extract on intestinal inflammation, intestinal barrier, brush border membrane functionality, intestinal microbiota, and morphology in vivo (*Gallus gallus* model). Cornish-cross fertile broiler eggs, at 17 days of embryonic incubation, were separated into groups as follows: non-injected (NI; this group did not receive an injection); 18 MΩ H_2_O (H_2_O; injected with ultrapure water), and 10 mg/mL (1%) chia phenolic extract (CPE; injected with phenolic extract diluted in ultrapure water). Immediately after hatch (21 days), chickens were euthanized and their small intestine, cecum, and cecum content were collected and analyzed. The chia phenolic extract reduced the tumor necrosis factor-alpha (TNF-α) and increased the sucrose isomaltase (SI) gene expression, reduced the *Bifidobacterium* and *E. coli* populations, reduced the Paneth cell diameter, increased depth crypt, and maintained villus height compared to the non-injected control group. Chia phenolic extract may be a promising beneficial compound for improving intestinal health, demonstrating positive changes in intestinal inflammation, functionality, microbiota, and morphology.

## 1. Introduction

Phenolic compounds, originating from plants’ secondary metabolism, possess significant functions in plant reproduction and growth while safeguarding against unfavorable environmental factors, pathogens, and herbivores. Moreover, these compounds contribute to several sensory attributes of plants, including color, flavor, and astringency. Their consumption has exhibited preventive effects against conditions such as obesity, cardiovascular disorders, diabetes, and neurodegenerative diseases [1,2,3,4].

Not every phenolic compound is absorbed in the small intestine; the majority of them make their way to the colon, where they act as a substrate for the resident microbiota. This interaction yields various effects, primarily by modulating the intestinal microbiota and promoting its homeostasis. Thus, phenolic compounds can be considered prebiotic substrates [5,6,7,8,9]. Compared to other compounds such as proteins, lipids, and carbohydrates, phytochemicals are not required for physiological functions; however, they have the capability to induce biological effects [5,10,11]. Prebiotic effects can occur by increasing the growth and establishment of probiotic bacteria and by reducing the number of pathogenic bacteria, such as *Escherichia coli*, *Helicobacter pylori*, and *Clostridium perfringens*. These impacts on the gut microbiota and beneficial properties for health occur through multiple means, including antioxidant, anti-inflammatory, bactericidal, immune, and mucus-producing functions [10,12,13,14]. Due to their bioavailability, the effects of phenolics are related to the food matrix, depending on whether they are consumed in the whole food or as isolated compounds [5,15,16,17]. However, the effects of isolating phenolic extracts on gut health, nutrient digestibility, absorption of some vitamins and minerals, and gut microbiota may differ depending on their dosage [1,18]. Therefore, phenolic compounds and other bioactive compounds from food sources need to have their effects and safety validated in in vivo studies.

Chia seed is rich in phenolic compounds, which are some of the main components with nutritional and functional activities of the seed, with a high antioxidant capacity [19,20,21,22]. According to some studies, the main phenolic compounds identified in chia were rosmarinic, caffeic, salvianolic, gallic, protocatechuic, ferulic acid, daidzin, glycitin, genistin, glycitein, and genistein acids [19,20,23,24]. A hydrolyzed phenolic extract of chia, in vitro, decreased the activity of gluconeogenic and glycolysis enzymes in HepG2 cells. These results suggest that phenolics from chia may reduce metabolic disorders related to obesity linked to impaired gluconeogenesis pathways [23]. Phenolic compounds may favor a diverse bacterial profile of the resident microbiota and promote colon health. The mechanisms of action by which phenolic compounds exert these effects are varied, but include actions such as regulating the expression of genes associated with inflammatory processes and preserving an adequate intestinal barrier [6,25,26].

Currently, the intra-amniotic administration model is widely used as an in vivo method to assess the effects of plant-derived bioactive compounds on gut health, including effects on intestinal brush border membrane (BBM) functionality and intestinal morphology. The *Gallus gallus* model contains a complex and dynamic intestinal microbiota, strongly influenced by host genetics, environment, and diet [27]. The intra-amniotic administration of black corn anthocyanin-rich extract promoted an improvement in the cecal microbiome while maintaining intestinal morphology and functionality [28]. The intra-amniotic administration of a hydrolyzed chia protein was evaluated and demonstrated that chia protein decreased tumor necrosis factor-alpha (TNF-α), increased the *Lactobacillus* population, and improved intestinal morphology, barrier, and functionality [29]; in addition, the intra amniotic administration of a soluble extract from chia improved the intestinal morphology and microbiota [24]. Therefore, the objective of this study was to evaluate the effects of the intra-amniotic administration of a chia phenolic extract on intestinal inflammation, intestinal barrier, the functionality of brush border membrane, intestinal microbiota, and morphology in vivo (*Gallus gallus* model).

## 2. Materials and Methods

### 2.1. Sample Material

#### 2.1.1. Chia Phenolic Extract

In this study, chia seeds (*Salvia hispanica* L.) cultivated in Brazil (specifically, in the state of Rio Grande do Sul) were utilized. The seeds were carefully packaged, transported in cardboard boxes, and subsequently stored in hermetically sealed plastic bags, shielded from light, and kept frozen at a temperature of −18 °C ± 1 °C until analysis. To obtain the chia flour, the seeds were ground, using a knife mill, in three separate replicates (Marconi Equipment, Piracicaba, Brazil).

For the extraction of chia phenolics, 20 g of chia flour was mixed with 100 mL of methanol:water (80:20, *v/v*), the mixture was placed in an ultrasonic water bath for 60 min at room temperature and then taken to centrifugation at 1792× *g* for 30 min. The supernatant was collected and taken to a rotatory evaporator at 50 °C, thus extracting the total phenolics. In sequence, acid hydrolysis by dissolution was performed, and the ratio of 1 mL of sample to 1 mL of HCl (2 M) was taken to a water bath at 80 °C for 60 min. Then, the extracted hydrolyzed phenolics were lyophilized, resulting in a dried extract.

#### 2.1.2. Determination of Total Phenolics

The total phenolic compounds was determined in the dried extract, in three replicates, using the Folin–Ciocalteau reagent [30]. The absorbance was measured in a spectrophotometer (Thermo Scientific, Evolution 606, Waltham, MA, USA) at 765 nm. Total polyphenols were expressed as milligrams of gallic acid equivalents per gram of sample (mg of GAE/g of sample).

**Figure 1:**
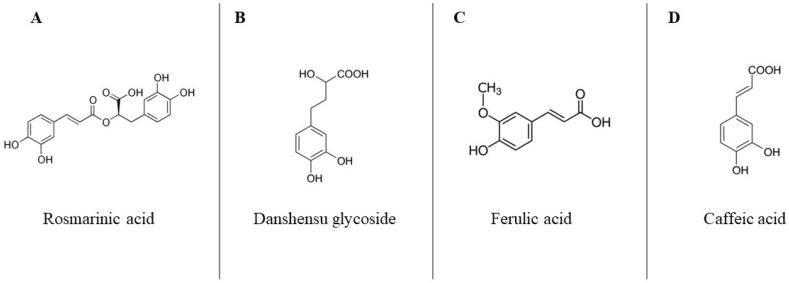
Chemical formulas of the major components of the chia phenolic extract. (**A**) Rosmarinic acid; (**B**) danshensu glycoside; (**C**) ferulic acid; and (**D**) caffeic acid.

A previous study conducted by our research group characterized the phenolic profile of a chia extract obtained from the same seed and using the same extraction procedure used in the present study [23]. The main phenolic compounds found in the extract were rosmarinic acid, danshensu glycoside, ferulic acid, and caffeic acid (Figure 1).

#### 2.1.3. Antioxidant Capacity

The antioxidant capacity was determined, in three replicates, using the radical scavenging activity assay using DPPH (1,1-diphenyl-2-picrylhydrazyl). Briefly, in a test tube, protected from light, lyophilized chia phenolic extract (100 µL) was added to an ethanolic DPPH solution and stirred by vortexing. After incubation (30 min), the absorbance was measured (517 nm) [31]. The antiradical activity was expressed in µmol of trolox equivalent per gram of sample (µmol of trolox equivalent/g of sample).

### 2.2. Intra-Amniotic Administration

Cornish-cross fertile broiler eggs (*n* = 27) were acquired from Moyer’s Chicks, a commercial hatchery located in Quakertown, PA, USA. The eggs were incubated under optimal conditions at the Cornell University Animal Science poultry farm incubator, with a temperature of 37 ± 2 °C and humidity of 89.6 ± 2%. The animal protocols followed in this study were approved by the Cornell University Institutional Animal Care and Use Committee, under the protocol code 2020-0077.

The chia phenolic extract was diluted in 18 MΩ H_2_O to determine the concentration. The dilution was intended to keep the osmolarity value (Osm) below 320 Osm, ensuring that the injection of the solution would not lead to dehydration in the chicken embryos. At 17 days of embryonic incubation, a candling process was performed to discard eggs that were cracked, infertile, contaminated, or contained early dead embryos. Eggs containing viable embryos were weighed and randomly divided into three groups, ensuring a similar weight distribution (*n* = 9/group). All eggs underwent disinfection with 70% ethanol. Subsequently, each group received an injection of the specified solution (1 mL per egg) using a 19 mm gauge needle. The injection was administered vertically into the amniotic fluid, which was identified through candling. The three groups were assigned as follows: non-injected (NI); 18 MΩ H_2_O (H_2_O); and 10 mg/mL (1%) chia phenolic extract (CPE). Once all the eggs had been injected, the injection sites were sealed using cellophane tape, and the eggs were arranged in hatching baskets in a way that ensured equal representation of each treatment at every incubator location. Immediately after hatch (21 days) and from each treatment group, the chickens were weighed and then euthanized by CO_2_ exposure. Subsequently, the small intestine (duodenum), cecum, and cecum content were collected from each chicken.

### 2.3. Extraction of the Total RNA from the Duodenum Samples

The RNA was extracted from 30 mg of the duodenum (*n* = 5 animals/group) according to the manufacturer’s protocol (RNeasy Mini Kit, Qiagen Inc., Valencia, CA, USA). All steps were carried out under RNase-free conditions. The total RNA was eluted in 50 µL of RNase-free water. The RNA was quantified by absorbance at 260/280, and the integrity of the 18S ribosomal RNAs was confirmed using 1.5% agarose gel electrophoresis, followed by ethidium bromide staining. Subsequently, the samples were frozen at (−80 °C) until analysis [28].

### 2.4. Real-Time Polymerase Chain Reaction (RT-PCR) and Prime Design

To obtain the cDNA, a total of 20 µL reverse transcriptase (RT) reaction was carried out in a BioRad C1000 touch thermocycler using the Improm-II Reverse Transcriptase Kit (Catalog #A1250; Promega, Madison, WI, USA). The concentration of the resulting cDNA was determined by measuring the absorbance at 260 nm and 280 nm, applying an extinction coefficient of 33 (for single-stranded DNA).

For gene expression analysis of the duodenum, a real-time polymerase chain reaction (RT-PCR) was conducted. The primers used in the real-time qPCR were designed based on gene sequences sourced from the Genbank database, utilizing the Real-Time Primer Design Tool software (https://www.idtdna.com/scitools/Applications/RealTimePCR/default.aspx) (IDT DNA, Coralvilla, IA, USA) [29]. The sequences and descriptions of the primers used can be found in Table 1. To assess primer specificity, a BLAST search against the genomic National Center for Biotechnology Information (NCBI) database was performed. The primer for *Gallus gallus* 18S rRNA was designed as a reference gene.

### 2.5. Real-Time qPCR Design

The procedures were conducted following previously described methods [24,32]. In summary, cDNA was utilized in 10 mL reactions, each containing 2 × BioRad SSO Advanced Universal SYBR Green Supermix (Hercules, CA, USA). To eliminate DNA contamination in the PCR mix, a “no template” control with nuclease-free water was included. For each reaction (in duplicates), 8 µL of the master mix and 2 µL of cDNA were pipetted into a 96-well plate. A standard curve with seven points in duplicates was assessed. The amplification of double-stranded DNA was carried out using the Bio-Rad CFX96 Touch (Hercules, CA, USA) under the following PCR conditions: initial denaturation at 95 °C for 30 s, followed by 40 cycles of denaturation at 95 °C for 15 s, annealing at various temperatures according to Integrated DNA Technologies (IDT) for 30 s, and elongation at 60 °C for 30 s. The gene expression data were obtained as Cp values using the “second derivative maximum” method, automatically computed using the Bio-Rad CFX Maestro 1.1 software (Version 4.1.2433.1219, Hercules, CA, USA). The real-time qPCR analysis included a standard curve, and a curve with four points was prepared through a 1:10 dilution (in duplicates). The software generated a graph of Cp values versus log 10 concentrations, and the efficiencies were calculated as 10 (1/slope). The specificity of the amplified real-time RT-PCR products was verified through melting curve analysis (60–95 °C) after 40 cycles, resulting in distinct specific products with their respective melting temperatures.

### 2.6. Collection of Microbial Samples and Intestinal Contents DNA Extraction

The cecum (*n* = 5 animals/group) was sterilely removed. The cecum contents were placed into a sterile 15 mL tube with 9 mL of sterile PBS and homogenized by vortexing with glass beads (4 mm diameter) for 3 min. Debris was separated by centrifugation at 1000× *g* for 5 min, and the resulting supernatant was collected and subjected to another centrifugation step at 4000× *g* for 10 min. The pellet obtained was washed twice with PBS and stored at −20 °C until DNA extraction. For DNA purification, the pellet was resuspended in 50 mM ethylenediaminetetraacetic acid (EDTA) and treated with lysozyme (Sigma Aldrich CO., St. Louis, MO, USA; final concentration of 10 mg/mL) for 45 min at 37 °C. The bacterial genomic DNA was subsequently isolated using the Wizard Genomic DNA purification kit (Promega Corp., Madison, WI, USA) [28].

### 2.7. Primer’s Design and PCR Amplification of Bacterial 16S rDNA

Primers for *Bifidobacterium*, *Lactobacillus*, *Escherichia coli*, and *Clostridium* were used, as established in previous studies [28,32]. Additionally, universal primers were designed to target the conserved region in the bacterial 16S rRNA and were utilized as internal standards. The PCR products were separated using 2% agarose gel, stained with ethidium bromide, and quantified using Quantity One 1-D analysis software version 4.6.8 (Bio-Ra, Hercules, CA, USA). The relative abundance of each examined bacterium was assessed. All products were normalized to the content of the universal 16S rRNA primer product, allowing for the determination of the proportions of each examined bacterial product.

### 2.8. Intestinal Morphology

Intestinal morphology assessments were conducted following previously established methods [24,29,32]. Duodenum samples were fixed in fresh 4% (*v/v*) buffered formaldehyde, dehydrated, cleared, and embedded in paraffin. Multiple 5 µm thick sections were cut and placed on glass slides. The sections were then deparaffinized in xylene and rehydrated using a series of graded alcohol solutions. Subsequently, the slides were stained with Alcian blue/periodic acid-Schiff and examined under a light microscope. The following morphometric measurements were assessed: villus height (µM), villus surface area (µM), crypt depth (µM), Paneth cell number and diameter (µM), goblet cell number and goblet cell diameter (µM) in both villi and crypts, as well as the characterization of goblet cell types (acidic, neutral, and mixed). Each biological sample (*n* = 3/treatment group) was subjected to examination in 4 segments, and within each segment, 10 randomly chosen villi and crypts were analyzed, resulting in 40 replicates per biological sample. The analysis was performed using a light microscope (CellSens Standard software, version 3.1, Olympus, Waltham, MA, USA).

For the Alcian blue and periodic acid-Schiff stain, the segments were only counted for the type of goblet cells (acid, neutral, or mixed) in both the villi and crypts. The count of goblet cells was performed on 10 villi and crypts per sample, and the means were calculated for statistical analysis. The villus surface area was determined using the following equation:

$$Villus\;surface\;area=2\frac{VW}{2}\times VL$$

where VW = villus width average of three measurements and VL = villus length.

### 2.9. Statistical Analysis

The experimental treatments were arranged in a completely randomized design, and the results were presented as means ± standard error deviation (SED). Statistical significance was determined at a *p*-value < 0.05. The normality of the data distribution was assessed using the Shapiro–Wilk normality test. For normally distributed data, differences between experimental groups were analyzed using a one-way analysis of variance (ANOVA), followed by a post-hoc Duncan test. In cases where the data did not follow a normal distribution, the Kruskal–Wallis test was applied, followed by a post hoc Dunn’s test. The correlation between intestinal parameters evaluated in our study was analyzed using Spearman’s correlation coefficient.

The statistical analyzes were carried out utilizing IBM SPSS Statistics^®^, version 25, while graphing was accomplished with GraphPad Prism^®^ version 9.0 software (GraphPad Software Inc., San Diego, CA, USA).

## 3. Results

### 3.1. Chia Phenolic Extract Characterization

The concentration of total phenolic compounds in chia phenolic extract was 405.17 mg of GAE/g of the sample, and the antiradical activity was 3.06 µmol of Trolox equivalent/g of the sample (Table 2).

### 3.2. Body Weight

Comparing all the groups, the body weight was similar, according to one-way ANOVA followed by the post hoc Duncan test. NI (35.00 ± 0.82), H_2_O (35.13 ± 1.09), and CPE (36.22 ± 0.88). Values are means in grams ± SED, *n* = 7–9/group (according to hatching).

### 3.3. Effect of Chia Phenolic Extract on Duodenal Gene Expression

**Figure 2:**
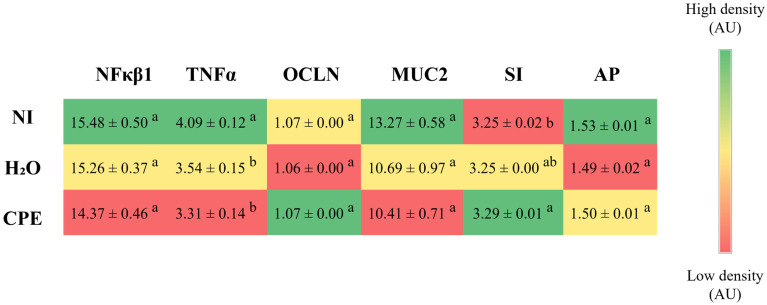
Effect of intra-amniotic administration of chia phenolic extract on intestinal gene expression. NI: non-injected; H_2_O: 18 MΩ H_2_O (water) injected; CPE: chia phenolic extract; NF-κβ1: nuclear factor-kappa beta; TNF-α: tumor necrosis factor-alpha; OCLN: occludin; MUC2: mucin 2; SI: sucrose isomaltase; AP: aminopeptidase; AU: arbitrary unit. Values are presented as means ± SED, *n* = 5/group. For each gene, within the same column, the color red indicates lower gene expression levels, whereas green indicates higher gene expression levels. ^a,b^ Per gene, within the same column, treatment groups with different letters indicate significant differences (*p* < 0.05) based on one-way ANOVA followed by post hoc Duncan test (for data with normal distribution) or according to Kruskal–Wallis and a post hoc Dunn’s test (for data without normal distribution).

The intra-amniotic administration of chia phenolic extract did not change nuclear factor-kappa beta (NF-κβ1) gene expression but reduced the tumor necrosis factor-alpha (TNF-α) gene expression compared to the non-injected control group. Regarding the intestinal barrier genes, occludin (OCLN) and mucin 2 (MUC2), chia phenolic extract maintained the expression of these genes. For the intestinal functionality genes, the chia phenolic extract increased the sucrose isomaltase (SI) gene expression compared to the non-injected control group and maintained the aminopeptidase (AP) gene expression (Figure 2).

### 3.4. Effect of Chia Phenolic Extract on the Bacterial Population on Cecum Content

**Figure 3:**
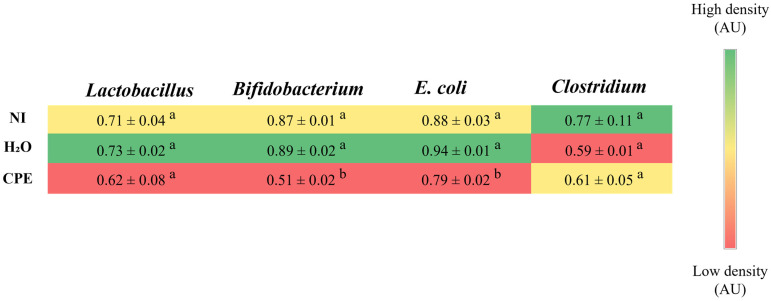
Effect of intra-amniotic administration of chia phenolic extract on the bacterial population on cecum content. NI: non-injected; H_2_O: 18 MΩ H_2_O (water) injected; CPE: chia phenolic extract; AU: arbitrary unit. Values are presented as means ± SED, *n* = 5/group. For each bacteria, within the same column, the color red indicates lower gene expression levels, whereas green indicates higher gene expression levels. ^a,b^ For each bacteria, within the same column, treatment groups with different letters indicate significant differences (*p* < 0.05) based on one-way ANOVA followed by post hoc Duncan test (for data with normal distribution) or according to Kruskal–Wallis and a post hoc Dunn’s test (for data without normal distribution).

The intra-amniotic administration of chia phenolic extract maintained the *Lactobacillus* population, reduced *Bifidobacterium* and *E. coli* populations compared to both control groups (non-injected and H_2_O), and maintained *Clostridium* populations (Figure 3).

### 3.5. Effect of Chia Phenolic Extract on Morphological Parameters in Duodenum

**Figure 4:**
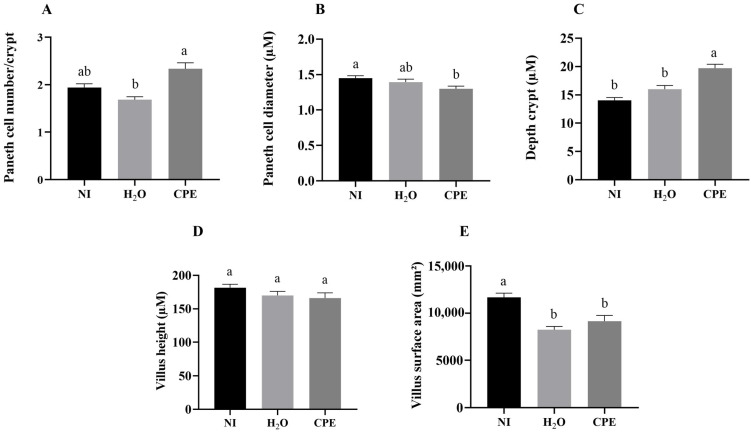
Effect of intra-amniotic administration of chia phenolic extract on morphological parameters in duodenum. NI: non-injected; H_2_O: 18 MΩ H_2_O (water) injected; CPE: chia phenolic extract; AU: arbitrary unit. (**A**) Paneth cell number/crypt; (**B**) Paneth cell diameter (µM); (**C**) depth crypt (µM); (**D**) villus height (µM); (**E**) villus surface area (mm^2^). Values are presented as means ± SED, *n* = 3 animals/group, 4 sections, 10 measurements. ^a,b^ Treatment groups with different letters indicate significant differences (*p* < 0.05) based on Kruskal–Wallis and a post hoc Dunn’s test.

The intra-amniotic administration of chia phenolic extract increased the Paneth cell number compared to the H_2_O control group and maintained compared to the non-injected control group. Paneth cell diameter was maintained compared to the H_2_O control group and reduced compared to the non-injected control group. The chia phenolic extract increased the depth crypt and maintained the villus height compared to both of the control groups (non-injected and H_2_O) but reduced the villus surface area compared to the non-injected control group (Figure 4).

The intra-amniotic administration of chia phenolic extract did not change the villus and crypt goblet cell diameter but reduced the goblet cell number in the crypt. For the villi goblet cells, the chia phenolic administration reduced the acidic and neutral types and increased the mixed goblet cells compared to the non-injected control group; for the crypt goblet cells, chia phenolic administration reduced all the types of goblet cells (acidic, neutral, and mixed) (Table 3).

### 3.6. Correlation Analysis

**Figure 5:**
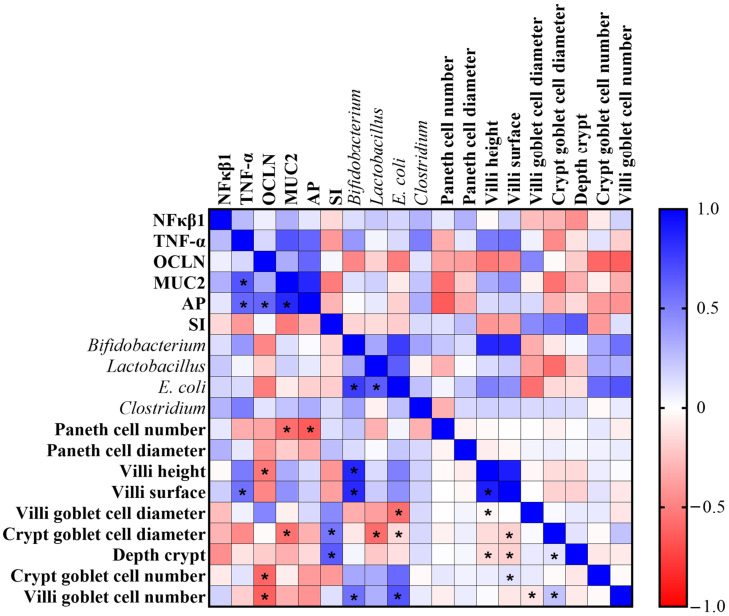
Heatmap demonstrating Spearman’s correlation analysis. NF-κβ1: nuclear factor-kappa beta; TNF-α: tumor necrosis factor-alpha; OCLN: occludin; MUC2: mucin 2; AP: aminopeptidase; SI: sucrose isomaltase. * Indicates a statistically significant difference (*p* < 0.05).

The correlation between parameters of intestinal health evaluated in this study was evaluated using Spearman’s correlation analysis. Positive correlations were observed between SI and depth crypt, *Bifidobacterium* and villus surface area, *E. coli* and crypt goblet cell number, and villus surface area and crypt goblet cell number, and a negative correlation was observed between villus surface area and depth crypt (Figure 5).

## 4. Discussion

Phenolic compounds can reach the colon, serving as a substrate for the resident microbiota; after they are absorbed and circulated in the body, they can exert a wide range of effects in the intestine, such as anti-inflammatory effects [1,33,34,35]. In this present study, we aimed to assess the impact of the intra-amniotic administration of a phenolic extract from chia seeds. We investigated its effects on the inflammatory response in the intestine, the intestinal barrier, the functionality of the brush border membrane, bacterial populations, and the overall morphology in vivo. Our results demonstrate that the phenolic compounds of chia reduced the TNF-α and increased the SI gene expression, reduced the *E. coli* population, reduced the Paneth cell diameter, and increased the Paneth cell number. Further, the chia phenolic extract increased the depth crypt and maintained the villus height in the *Gallus gallus* model. These effects might occur based on the concentration of total phenolics and antioxidant capacity (Table 2) found in the extract.

In the present study, the administration of a chia phenolic extract downregulated the TNF-α gene expression compared to the non-injection control group (Figure 2). TNF-α is an activator of the production of different cytokines, promoting a pro-inflammatory state. In general, long-term elevated TNF-α levels are associated with conditions such as inflammatory bowel disease, and lowering the levels of this cytokine is generally considered to have a beneficial anti-inflammatory effect [36,37,38]. A previous study characterized the chia phenolic extract and found that the main phenolic compounds in the extract were rosmarinic acid, danshensu glycoside, ferulic acid, and caffeic acid [23]. The study by Lu et al., (2022) [39] showed that treatment with rosmarinic acid inhibited the increase in TNF-α, IL-6, and IL-8 in the liver, which indicated that rosmarinic acid could suppress the inflammatory response by inhibiting inflammatory cytokines. Furthermore, the administration of an extract rich in rosmarinic acid decreased TNF-α in mice fed a high-fat diet [40]. Danshensu was demonstrated to be an efficient antioxidant and also had an effective anti-inflammatory impact [41,42], whereas ferulic acid increased antioxidant capacity, improved intestinal barrier integrity, mucosal immune response, and increased stability of ileal microflora [43]. Moreover, it is proposed that the digestion process of chia products could lead to an elevation in antioxidant activity and favor the anti-inflammatory effects [44]. Thus, the phenolic composition of the extract with anti-inflammatory action might justify the reduction in TNF-α gene expression.

The expression of the SI gene was found to be increased in the group that received the chia phenolic extract injection (Figure 2). Sucrase isomaltase (SI) is a disaccharidase found in the brush border membrane of the small intestine, responsible for breaking down disaccharides or oligosaccharides into monosaccharides for absorption. This enzyme plays a crucial role in the digestion of starchy foods and foods containing sugars [45]. A previous study by Enes et al., (2020) demonstrated that, in vitro, a chia phenolic extract downregulated mRNA of enzymes involved in gluconeogenesis and glycolysis (phosphoenolpyruvate carboxykinase and glucose 6-phosphatase, and phosphofructokinase and pyruvate kinase, respectively) [23]. Simsek et al., (2017) [46] demonstrated that caffeic acid, which is found in the phenolic extract of chia [23], induced upregulation of jejunal SI mRNA expression, and suggested that such regulatory effects by dietary compounds, such as phenolics, might be linked to the mechanisms of action needed to further demonstrate potential pharmacological properties. Caffeic acid from propolis improved glucose uptake and decreased glucose 6-phosphatase expression in insulin resistance in vitro; it also improved hyperglycemia, glucose tolerance, and hyperlipidemia, and reduced TNF-α expression in vivo [47]. Moreover, other chia phenolics may also have provided this effect, such as rosmarinic acid, which has been associated with glucose improvement related to AMPK phosphorylation [48]. The upregulation of SI mRNA expression can be a mechanism for glucose metabolism regulation by phenolic compounds, but more studies are needed to better elucidate the pathway.

Despite the reduction in *Bifidobacterium*, the chia phenolic extract reduced *E. coli* and maintained *Clostridium* and *Lactobacillus* populations compared to non-injected and H_2_O groups (Figure 2). Increased *E. coli* has been found in pathologies such as celiac disease, non-alcoholic fatty liver disease, inflammatory bowel disease, and gastrointestinal cancer [15]. The ability of polyphenols to reduce *E. coli* has been previously reported [49,50,51], and some examples are the polyphenols of red wine [52], gallic acid [53], and grape pomace [54]. An extract of black corn, rich in anthocyanin, demonstrated in ovo a result similar to ours, reducing the *E. coli* and maintaining the *Lactobacillus* population [28]. Variations in the *Lactobacillus* population in our in ovo studies seem to be more sensitive to the presence of fiber-rich soluble extracts [24,32]. The precise mechanisms through which polyphenols regulate the microbiota are not fully understood, but, in general, they possess the ability to intervene both directly and indirectly. The inhibition of certain bacteria is related to the antimicrobial capacity of these compounds. Some phenolics can inhibit the growth of bacteria that are harmful to health, reducing cell adhesion between them and the intestinal epithelium, and negatively regulating genes that encode bacteria’s membrane proteins. Polyphenols can also act indirectly, influencing the growth of some bacteria which, in turn, modulate the development of others [15,55].

The chia phenolic extract intra-amniotic administration increased the number of Paneth cells and reduced the diameter of Paneth cells (Figure 4A,B). In this way, an improvement in Paneth cell development without altering the cell size was achieved. The antimicrobial peptides secreted by Paneth cells were not produced, as there were no triggering factors such as inflammation or pathogenic bacteria, which is in accordance with our results about the reduction in TNF-α and *E. coli*. Similar results were verified after the intra-amniotic administration of a hydrolyzed protein of chia seed [29]. The intra-amniotic administration of chia phenolic extract increased the depth crypt (Figure 4C). In the intestine, the increase in the height of villi and the depth of crypts directly affects the ability to absorb nutrients, as it increases the absorptive area [56], which is in accordance with the positive correlation we found between SI and depth crypt. In our study, the chia phenolic extract administered could increase the depth of the crypts and maintain the villi height (Figure 4D). The chia phenolic extract reduced the villus surface area compared to the non-injected group (Figure 4E). This effect was also observed with the intra-amniotic administration of grape pomace [57]. The findings were attributed to the ability of phenolic compounds to interact with proteins and form indigestible complexes. As proteins are crucial for cell division and proliferation, the interaction between polyphenols and proteins might decrease protein digestibility, which could explain the decrease in duodenal enterocyte proliferation and, consequently, the reduction in villus surface area [57].

The goblet cell is a type of secretory enterocyte, from which intestinal MUC2 and mucus originate. MUC2 tends to show a positive correlation with the number and function of goblet cells, making it a specific marker indicative of goblet cell secretory activity. [58]. Our results showed that the intra-amniotic administration of a chia phenolic extract was not able to increase the number of goblet cells in the crypt (Table 3). This justifies the fact that there was no increase in MUC2 gene expression, and it is correlated with the reduction in *E. coli* (Figure 5). The same correlation (positive correlation between *E. coli* and crypt goblet cell number) was observed by Verediano et al., 2022 [28], who evaluated the intra-amniotic administration of a black corn anthocyanin-rich extract.

Chia is rich in dietary fiber, linolenic acid, protein, and bioactive compounds, such as phenolics, which are some of the main components with nutritional and functional activities of the seed [19,20,21]. Chia has a nutritional composition that suggests promising effects on intestinal health. Therefore, our research group has been evaluating the effects of chia and its fractions on intestinal health. Studies evaluating chia flour demonstrated that the intake of chia increased the production of short-chain fatty acids and improved the intestinal morphology in male *Wistar* rats [59], ovariectomized female *Wistar* rats [60], and male *Wistar* rats with metabolic disorders caused by the consumption of a high-fat high fructose diet [61]. In addition, the chia flour intake reduced the intestinal pH [60,61] and increased microbiota richness [59,60]. When we evaluated the chia fractions, the intra-amniotic administration of a hydrolyzed chia protein downregulated TNF-α expression, increased the gene expression of OCLN, MUC2, and AP, and improved the morphology [29]. The intra-amniotic administration of a soluble extract from chia, rich in fiber, improved the intestinal morphology, increased the relative abundance of *Bifidobacterium* and *Lactobacillus*, and upregulated the expression of proteins related to mineral metabolism [24]. In this way, in the present study, we aimed to evaluate the effects of a methanolic extract of chia, rich in phenolic compounds, on intestinal health in the *Gallus gallus* model. The consumption of chia flour, the intra-amniotic administration of a soluble extract from chia seed, and a hydrolyzed protein of chia demonstrated more effects on intestinal morphology than the intra-amniotic administration of an isolated phenolic extract. In this sense, it is important to consider the relevance of nutrient synergy, as we can see with the intake of chia flour, so that all compounds (dietary fibers, proteins, and bioactive compounds) are ingested from the same food matrix. Furthermore, the administration of nutrients required for physiological functions (such as fiber and protein) along with phenolic compounds can have a greater impact on intestinal morphology, especially in a naive organism with a developing gastrointestinal tract. The presence of high concentrations of phenolic compounds in food does not always guarantee a high bioaccessibility of these phenolics. The beneficial effects of phenolics are also influenced by other factors such as stability, the composition of the microbiota, and the activity of digestive enzymes [15,62]. Biotransformations mediated by the intestinal microbiota play a role in determining the bioavailability of phenolics, which is further influenced by processes of absorption and metabolism [15,63]. Therefore, in a complex diet with the ingestion of several types of food, it is important to consider how phenolics are ingested in the diet, such as whether they are present in food, in which they will relate to other bioactive compounds and nutrients, or in an isolated form. A different dosage of phenolic compounds might be needed to exert more effects, and/or a more appropriate form of administration (in food, isolated, microencapsulated). In this way, more, and longer, in vivo studies at different dosages and different forms of administration are needed to improve knowledge about the effects of phenolic compounds from chia. The introduction of the use of extracts in medical practice is restricted due to the lack of possibility of standardization and control of the constancy of the extract composition; however, research in this area is essential to elucidate questions regarding bioactives and associated potential beneficial effects on intestinal functionality and the microbiome, and in order to guide future efforts towards enabling the use of these compounds as nutraceuticals.

## 5. Conclusions

The intra-amniotic administration of chia phenolic extract improved markers related to inflammation, intestinal functionality, bacterial population, and morphology by reducing TNF-α, increasing the SI gene expression, reducing the *E. coli* population, and increasing depth crypt in the *Gallus gallus* model. Chia phenolic extract may be a promising beneficial compound for improving intestinal health, and further studies are needed to better elucidate the most appropriate form of administration (in food, isolated, microencapsulated) and the dosage.

## Figures and Tables

**Table 1:** Sequence of primers used in this study.

Gene	Oligonucleotides (5′-3′)	
	Forward Primer (5′-3′)	Reverse Primer (5′-3′)
BBM functionality		
AP	CGTCAGCCAGTTTGACTATGTA	CTCTCAAAGAAGCTGAGGATGG
SI	CCAGCAATGCCAGCATATTG	CGGTTTCTCCTTACCACTTCTT
18S rRNA	GCAAGACGAACTAAAGCGAAAG	TCGGAACTACGACGGTATCT
Inflammation		
TNF-α	GACAGCCTATGCCAACAAGTA	TTACAGGAAGGGCAACTCATC
NF-κβ1	CACAGCTGGAGGGAAGTAAAT	TTGAGTAAGGAAGTGAGGTTGAG
Intestinal barrier		
MUC2	CCTGCTGCAAGGAAGTAGAA	GGAAGATCAGAGTGGTGCATAG
OCLN	GTCTGTGGGTTCCTCATCGT	GTTCTTCACCCACTCCTCCA

BBM: brush border membrane; AP: aminopeptidase; SI: sucrose isomaltase; 18S rRNA: reference gene; TNF-α: tumor necrosis factor-alpha; NF-κβ1: nuclear fator-kappa beta; MUC2: mucin 2; OCLN: occludin.

**Table 2:** Characterization of chia phenolic extract.

Variable	Mean ± SD
Total phenolic compounds (mg of GAE/g of sample)	405.70 ± 17.58
Antiradical activity (µmol of Trolox equivalent/g of sample)	3.06 ± 0.05

GAE: gallic acid equivalent; SD: standard deviation; total phenolic compounds and antiradical activity were analyzed in three replicates, by spectrophotometry.

**Table 3:** Effect of chia phenolic extract on goblet cells.

	NI	H_2_O	CPE
Villi goblet cell diameter (µM)	2.46 ± 0.06 ^a^	2.20 ± 0.05 ^a^	2.64 ± 0.08 ^a^
Crypt goblet cell diameter (µM)	2.92 ± 0.05 ^b^	3.13 ± 0.05 ^a^	2.96 ± 0.06 ^ab^
Villi goblet cell number	24.68 ± 0.74 ^b^	38.38 ± 0.91 ^a^	23.78 ± 0.60 ^b^
Acidic	15.28 ± 0.71 ^b^	26.71 ± 1.12 ^a^	11.73 ± 0.65 ^c^
Neutral	0.79 ± 0.13 ^a^	0.10 ± 0.04 ^c^	0.50 ± 0.11 ^b^
Mixed	8.68 ± 0.57 ^b^	11.57 ± 0.66 ^a^	11.55 ± 0.46 ^a^
Crypt goblet cell number	12.67 ± 0.55 ^a^	10.95 ± 0.62 ^b^	9.33 ± 0.35 ^b^
Acidic	8.53 ± 0.43 ^a^	7.88 ± 0.51 ^ab^	6.99 ± 0.28 ^b^
Neutral	0.41 ± 0.06 ^a^	0.50 ± 0.07 ^a^	0.10 ± 0.03 ^b^
Mixed	3.73 ± 0.27 ^a^	2.58 ± 0.21 ^b^	2.25 ± 0.16 ^b^

Values are presented as means ± SED, *n* = 3 animals/group, 4 sections, 10 measurements. NI: non-injected; H_2_O: 18 MΩ H_2_O (water) injected; CPE: chia phenolic extract. ^a–c^ Treatment groups with different letters indicate significant differences (*p* < 0.05) based on Kruskal–Wallis and a post hoc Dunn’s test.

## Data Availability

Not applicable.
